# Benign Metastasizing Leiomyoma of the Uterus: Rare Manifestation of a Frequent Pathology

**DOI:** 10.1155/2018/5067276

**Published:** 2018-10-30

**Authors:** Maria Inês Raposo, Catarina Meireles, Mariana Cardoso, Mariana Ormonde, Cristina Ramalho, Mónica Pires, Mariana Afonso, Almerinda Petiz

**Affiliations:** ^1^Department of Gynecology, Francisco Gentil Portuguese Oncology Institute, Porto, Portugal; ^2^Department of Gynecology, Hospital of Divino Espírito Santo of Ponta Delgada, EPER, São Miguel, Azores, Portugal; ^3^Department of Pathology, Francisco Gentil Portuguese Oncology Institute, Porto, Portugal

## Abstract

Benign Metastasizing Leiomyoma (BML) is a rare condition with few cases reported in the literature. It is usually incidentally diagnosed several years after a primary gynecological surgery for uterine leiomyoma. Differential diagnosis of BML is complex requiring an extensive work-up and exclusion of malignancy. Here, we report two cases of BML based on similarity of histopathological, immunohistochemical, and genetic patterns between lung nodules and uterine leiomyoma previously resected, evidencing the variability of clinical and radiological features of BML. We highlight the importance of 19q and 22q deletions as highly suggestive of BML. These findings are particularly relevant when there is no uterine sample for review.

## 1. Introduction

Uterine leiomyoma is the most common gynecological tumor [1–4]. BML is a rare variant [1–13] characterized by multiple leiomyomatous lesions in distant locations, most commonly the lungs [3–11]. Less frequently involved areas are lymph nodes, inferior vena cava, heart, brain, bones, abdomen, retroperitoneum, pelvis, breast, esophagus, liver, appendix, trachea, skin, muscle, and parametria [14, 15]. The antagonistic terminology of BML reflects the coexistence of benign appearance with a biological behavior suggesting malignancy [2, 3, 8, 16]. When multiple pulmonary nodules are incidentally detected in women with history of surgery for uterine leiomyoma, clinicians should be aware of this potential diagnosis [5, 11, 13].

The incidence of BML remains unclear [6]. Since its first publication by Steiner, in 1939 [18], approximately 150 cases have been published [1]. Due to the rarity of this condition, the pathophysiology and management remains controversial [2, 4]. The literature is scarce on studies regarding the cytogenetic evaluation [7].

Here we report two clinical cases of BML diagnosed in the Portuguese Oncology Institute of Porto (Table 1). Our aim is to review its diagnostic challenges, focusing on clinical, radiological, and anatomopathological findings. We also intend to determine the implications of cytogenetic study of this rare condition.  Table 2 summarizes the case reports regarding pulmonary BML recently published in the literature.

## 2. Case Presentation

### 2.1. Case 1

**Figure 1:**
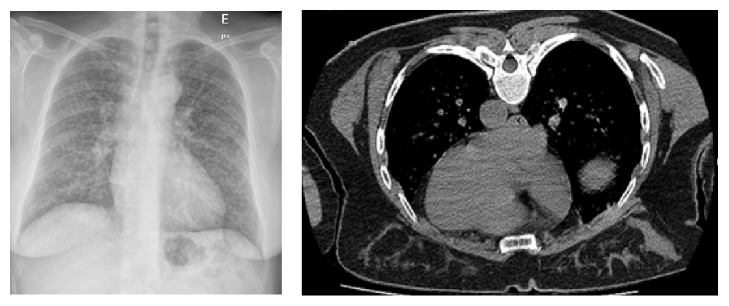
Chest radiography and CT images of patient 1.

A 49-year-old, premenopausal, asymptomatic woman, with past clinical history significant for total hysterectomy 10 years earlier due to a leiomyoma of the uterus, presented with a miliary pattern in a routine chest radiography as in computed tomography (CT) scan (Figure 1). We performed a Positron Emission Tomography (PET) scan that showed weak fluorodeoxyglucose (FDG) uptake in lung nodules. She underwent CT-guided biopsy of a pulmonary nodule which revealed spindle cells consistent with smooth muscle differentiation, without cellular atypia, necrosis, or mitotic figures. Immunohistochemical examination was positive for smooth muscle actin (SMA), desmin, estrogen, and progesterone receptors and was negative for HBM-45, CK7, and S100. The proliferative index, assessed with Ki-67 index, was low. Cytogenetic evaluation of lung tumor tissue showed 19q and 22q terminal deletions. Cytogenetic analysis of previous leiomyoma was not performed due to insufficient pathological material. After diagnosing BML, patient underwent bilateral salpingo-oophorectomy followed by Letrozole therapy. At 9 months follow-up, there was no further development of the disease.

### 2.2. Case 2

**Figure 2:**
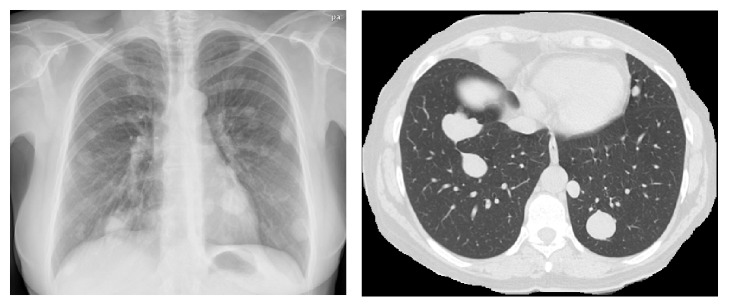
Chest radiography and CT images of patient 2.

**Figure 3:**
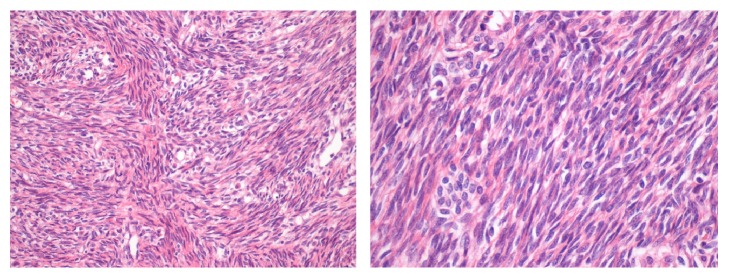
Histopathologic examination of BML and uterine leiomyoma of patient 2.

**Figure 4:**
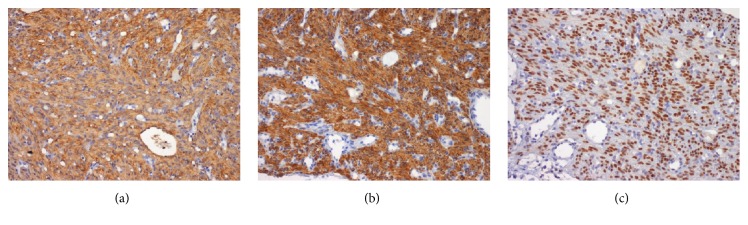
Immunohistochemical staining of BML and uterine leiomyoma of patient 2. (a) SMA, (b) Desmin, and (c) Hormonal Receptors.

**Figure 5:**
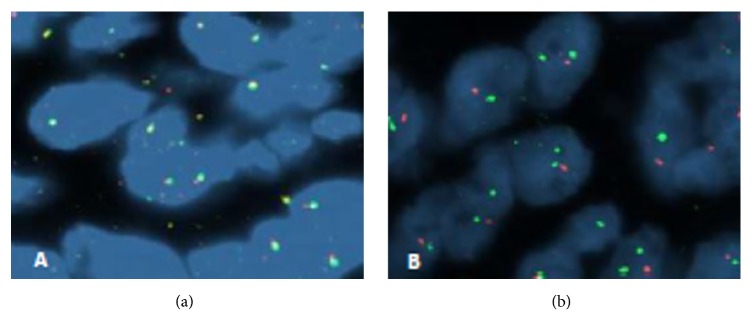
Cytogenetic study of BML and uterine leiomyoma of patient 2, using “*Fluorescence In Situ Hybridization” (FISH), *with probes* LSI EWSR1 (22q12) Dual Color, Break Apart Rearrangement Probe, Abbott*, and* ZytoLight SPEC 19q13/19q13 Dual Color Probe, Zytovision. (a) *22q12 deletion and (b) 19q13 deletion.

A 48-year-old premenopausal woman was referred because of persistent cough. Her past clinical history included a hysterectomy 13 years earlier for uterine leiomyoma. Chest radiography and CT revealed multiple pulmonary bilateral nodules (Figure 2) with no FDG uptake in the PET scan. CT-guided biopsy of a pulmonary nodule was performed and the resected uterine leiomyoma was reviewed. Both specimens showed identical histopathology of a low grade, benign appearing, and smooth muscle tumor (Figure 3). The immunohistochemical profile of BML is indistinguishable from that of the primary uterine tumor with positivity for SMA, desmin, estrogen, and progesterone receptors (Figure 4) and negativity for HMB-45, CD31, CD34, and EMA. The staining for ki-67 showed low mitotic activity. Cytogenetic analysis revealed shared profile between both samples, including 19q and 22q terminal deletions (Figure 5). Since these findings were consistent with BML, surgical castration was performed. After 6 months of follow-up, the remaining lesions were stable.

## 3. Discussion

BML is found primarily in reproductive aged women [2, 5, 11], as in the presented cases. The mean age at diagnosis is 47,3 years [6]. The course of the disease correlates with the level of reproductive hormones [5]. Several theories have been proposed along the years regarding the etiology of BML, including [5–10] hematogenous spread of uterine leiomyoma;* in situ* proliferation of smooth muscle induced by hormonal stimulation; metastasis of low-grade uterine leiomyosarcoma previously subdiagnosed; peritoneal seeding after surgery for uterine leiomyoma and metaplastic transformation. Since most cases of BML occur from 8,8 to 15 years after gynecological surgery [2, 5, 6, 8], we hypothesize that surgically induced vascular spread is the most likely cause [1, 10]. In addition, we agree with the majority of researchers who consider that BML is clonally derived from uterine leiomyoma [3–8]. The exclusive occurrence in women with history of uterine leiomyoma, the positivity for hormonal receptors, and the susceptibility to antihormonal therapy favor this origin [4, 13]. Overlapping in histopathological, immunohistochemical, and cytogenetic findings between pulmonary and uterine lesions suggests their association [6, 7].

Main clinical symptoms of BML vary depending on the organs involved [15]. Regarding pulmonary BML, patients are usually asymptomatic [2, 5, 8, 13] and the disease is an incidental finding, as we described in patient 1. Only one-third of patients develop respiratory symptoms, such as cough, hemoptysis, dyspnea, thoracalgia, and respiratory failure [3]. Hemothorax and pneumothorax have also been reported [4, 6].

Imaging findings are not specific for pulmonary BML [4, 5]. Multiple bilateral well-circumscribed pulmonary nodules are found in the majority of patients [1, 2, 8]. Another rarely reported features are solitary pulmonary nodule, interstitial lung disease, cystic lesions, cavitary lung nodules, and miliary pattern [5, 8, 11]. Radiologic findings of extrapulmonary BML are rarer and less well characterized in the literature. However, both pulmonary and extra-pulmonary nodules of BML show weak or absent FDG uptake on PET [15]. This allows exclusion of metastasis from uterine sarcoma or extrathoracic malignant tumors [1, 2].

Histopathological confirmation is required for definitive diagnosis of BML [1, 5]. These lesions reveal a smooth muscle phenotype with low mitotic activity, limited vascularization and lacks of anaplasia and necrosis [4–8, 10–13]. Its immunohistochemical features include positivity for smooth muscle actin, desmin, caldesmon, calponin, vimentin [1, 2], and hormonal receptors (estrogen and progesterone receptors) [1, 5]. Low ki-67 index [1, 5] and negativity for HMB-45 [4] are useful for ruling out uterine leiomyosarcoma and lymphangioleiomyomatosis, respectively. It is extremely important to differentiate BML from uterine leiomyosarcoma since follow-up and treatment are distinct [5, 8, 13].

Recent genetic studies confirm a shared profile between BML and uterine tumor [6, 7, 12]. The present study contributes to the individualization of BML as a genetically distinct entity, since both patients had 19q and 22q terminal deletions in pulmonary tissue, as previously described by* Nucci M. et al. *[12]. This cytogenetic profile was found in 3% of uterine leiomyomas, suggesting that BML arises from a biologically distinct minority of leiomyomas [12, 14]. Consequently, these mutations could be used as a marker for uterine leiomyomas with potential to develop BML. Given the rarity of this disease, we do not recommend performing a genetic screening test for all women undergoing surgery due to uterine leiomyoma [6]. However, from our standpoint, searching for 19q and 22q terminal deletions in lung nodules of women with past history of gynecological surgery has a determinant role in the differential diagnosis of BML. Therefore, this genetic study becomes even more useful for BML diagnosis when uterine specimen is unavailable or insufficient for retrospective review [12].

Since BML treatment is not standardized [1, 2, 4] it should be individualized for each patient depending on the metastasis sites [17]. If the disease is resectable,* en bloc* removal of lesions should be attempted [15, 16]. For pulmonary BML, although primary option consists in surgical excision of the maximum possible number of pulmonary nodules, it may not be technically feasible. Alternative therapies include surgical castration by bilateral oophorectomy, chemical castration [1, 2, 5, 10, 11], or combined therapy [2]. Some researchers advocate expectant treatment in climacteric women [2]. BML usually has an indolent evolution [2, 4] and favorable prognosis [11, 13]. According to the literature, after the excision of intrapulmonary lesions the median survival rate is 94 months [3, 13]. The patients described were re-examined every three months using a pulmonary CT. Although their pulmonary lesions remained stable, an extended follow-up is required to track disease progression.

## 4. Conclusion

A multidisciplinary approach is crucial for the diagnosis of BML in women with pulmonary smooth muscle neoplasia and history of uterine leiomyoma. The striking resemblance of BML to uterine fibroids should lead to correct diagnosis. When primary uterine tumor cannot be reassessed, the presence of 19q and 22q terminal deletions in lung nodules is strongly predictive of BML [10, 12], promoting proper treatment and surveillance for this benign condition. In the future, new cytogenetic markers may optimize BML diagnosis [7, 9]. Further studies are necessary to clarify the etiology of BML and standardize its management.

## Figures and Tables

**Table 1:** Clinical cases.

**Case**	**Age**	**Respiratory symptoms**	**Primary surgery for leiomyoma**	**Radiology**	**Final diagnosis**	**Microscopy and Immunohistochemistry**	**Cytogenetic evaluation**	**Treatment**	**Follow-up**
1	49	Asymptomatic	Total hysterectomy, 10 years ago	Miliary pattern PET: weak FDG uptake	CT-guided biopsy	Smooth muscle tumor, SMA+, desmin +, hormonal receptors+, low Ki-67	Lung tumor: 19q13 and 22q12 deletions	Bilateral salpingo-oophorectomy and Letrozole.	9 months, stable

2	48	Cough	Total hysterectomy, 13 years ago	Multiple pulmonary nodules PET: weak FDG uptake	CT-guided biopsy	Smooth muscle tumor, SMA+, desmin +, hormonal receptors+, low ki-67	Lung tumor and primary leiomyoma: 19q13 and 22q12 deletions	Bilateral salpingo-oophorectomy	6 months, stable

PET= positron emission tomography; FDG= Fluorodeoxyglucose; CT= computed tomography; SMA=smooth muscle actin.

**Table 2:** Pulmonary BML case reports.

**Refer**	**Age**	**Respiratory symptoms**	**Primary surgery for leiomyoma**	**Radiology**	**Final diagnosis**	**Microscopy and Immunohistochemistry**	**Cytogenetic evaluation**	**Treatment**	**Follow-up**
Nurettin et al. [1]	41	Dyspnea	Myomectomy, 10 years ago	Multiple pulmonary nodules PET: no FDG uptake	VATS biopsy	Smooth muscle tumor, SMA+, desmin +, hormonal receptors+, low ki-67	Not applicable	Bilateral salpingo-oophorectomy, total hysterectomy and Progesterone	5 years, stable

Ma et al. [3]	45	Asymptomatic	Myomectomy, 11 years ago	Multiple pulmonary nodules PET: abnormal FDG uptake	Aspiration Biopsy	Smooth muscle tumor, SMA+, desmin +, hormonal receptors+, ki-67=1%	Not applicable	Pulmonary wedge resection	5 months, stable

Chen et al. [5]	32	Chest tightness and labored breathing	Myomectomy, 1 month earlier	Miliary nodules	Thoracoscopic Biopsy	Spindle cells, SMA+, desmin +, hormonal receptors+	Not applicable	Tamoxifen	3 months, stable

Lee et al. [8]	52	Asymptomatic	Vaginal hysterectomy, 10 years ago	Multiple lung cavitations and nodules PET: no FDG avid	Needle Biopsy	Spindle cells, SMA+, desmin +, hormonal receptors+	Not applicable	GnRH Agonist	15 months, stable

Ras et al. [9]*∗*	53	Asymptomatic	Myomectomy, 26 years earlier	Multiple pulmonary nodules	Thoracotomy Biopsy	Bland smooth muscle cells, desmin +, hormonal receptors+, low ki-67	Not applicable	Subtotal hysterectomy, bilateral salpingo-ooforectomy, removal of the tumors from parametria and appendectomy and pulmonary wedge resection by thoracotomy	Not applicable

Ottlakan et al. [10]	36	Asymptomatic	Hysterectomy, 7 years earlier	Multiple pulmonary nodules	Core Biopsy	Smooth muscle cells, SMA+	Lung nodules: 19q22q deletion	Pulmonary wedge resection and cautery resection, through mini-thoracotomy (seven procedures)	Many recurrences

Patré et al. [11]*∗*	76	Acute respiratory distress	Total hysterectomy, 4 years earlier	Multiple pulmonary nodules and pleural effusion	Surgical biopsy	Spindle cells, SMA+, desmin +, hormonal receptors+, caldesmon+	Not applicable	Resection of pulmonary nodules, removal of trochanteric lesion and aromatase inhibitors	45 months, stable

Khan et al. [14]	47	Shortness pf breath and chest pain	Cervical hysterectomy, 3 years prior	Multiple pulmonary nodules PET: mild FDG uptake	CT guided biopsy and VATS biopsy	Smooth muscle tumor, SMA+, desmin +, hormonal receptors+, HMB45-, CD34-, EMA-	Lung nodules: Loss of 19 and 22 and deletion of 1p	VATS wedge resection and anastrozole	12 months, stable

Bakkensen et al. [15]*∗*	46	Asymptomatic	Total hysterectomy, 7 years ago	Multiple pulmonary nodules PET: no FDG uptake	CT guided biopsy	Bland spindle cells, SMA+, desmin +, hormonal receptors+	Not applicable	Bilateral salpingo-ooforectomy, resection of pelvic mass, opportunistic appendectomy and letrozole	2 years, stable

Zhong et al. [17]*∗*	51	Asymptomatic	Myomectomy, 26 years earlier	Multiple pulmonary nodules PET: abnormal FDG uptake	CT guided biopsy	Spindle-shaped cells, SMA+, desmin+, hormonal receptors+, CD34-, S100-, HMB45-, Ki-67<20%	Not applicable	Removal of lumbar spine tumor and Tamoxifen	5 months, stable

*∗*BML of other sites; PET= positron emission tomography; FDG= fluorodeoxyglucose; VATS=video-assisted thoracoscopic surgery; CT= computed tomography; SMA=smooth muscle actin.
